# Participatory learning and action cycles with women’s groups to prevent neonatal death in low-resource settings: A multi-country comparison of cost-effectiveness and affordability

**DOI:** 10.1093/heapol/czaa081

**Published:** 2020-10-21

**Authors:** Anni-Maria Pulkki-Brännström, Hassan Haghparast-Bidgoli, Neha Batura, Tim Colbourn, Kishwar Azad, Florida Banda, Lumbani Banda, Josephine Borghi, Edward Fottrell, Sungwook Kim, Charles Makwenda, Amit Kumar Ojha, Audrey Prost, Mikey Rosato, Sanjit Kumer Shaha, Rajesh Sinha, Anthony Costello, Jolene Skordis

**Affiliations:** 1 Department of Epidemiology and Global Health, Umeå University, Umeå S-901 87, Sweden; 2 UCL Institute for Global Health, UCL (University College London), 30 Guilford Street, London, WC1N 1EH, UK; 3 Perinatal Care Project, Diabetic Association of Bangladesh, 122 Kazi Nazrul Islam Avenue, Dhaka 1000, Bangladesh; 4 MaiMwana Project, Mchinji, Malawi; 5 Parent and Child Health Initiative (PACHI), Area 14 Plot 171, Lilongwe, Malawi; 6 Department of Global Health and Development, London School of Hygiene and Tropical Medicine, 15-17 Tavistock Place, London, WC1H 9SH, UK; 7 Warwick Medical School, University of Warwick, Coventry, CV4 7AL, UK; 8 Ekjut, Plot no. - 556B, Potka Chakradharpur, West Singhbhum, Pin - 833102, Jharkhand, India; 9 Women and Children First (UK), United House, North Road, London, N7 9DP, UK

**Keywords:** Costs, cost-effectiveness analysis, randomized controlled trial, maternal and child health, community mobilization

## Abstract

WHO recommends participatory learning and action cycles with women’s groups as a cost-effective strategy to reduce neonatal deaths. Coverage is a determinant of intervention effectiveness, but little is known about why cost-effectiveness estimates vary significantly. This article reanalyses primary cost data from six trials in India, Nepal, Bangladesh and Malawi to describe resource use, explore reasons for differences in costs and cost-effectiveness ratios, and model the cost of scale-up. Primary cost data were collated, and costing methods harmonized. Effectiveness was extracted from a meta-analysis and converted to neonatal life-years saved. Cost-effectiveness ratios were calculated from the provider perspective compared with current practice. Associations between unit costs and cost-effectiveness ratios with coverage, scale and intensity were explored. Scale-up costs and outcomes were modelled using local unit costs and the meta-analysis effect estimate for neonatal mortality. Results were expressed in 2016 international dollars. The average cost was $203 (range: $61–$537) per live birth. Start-up costs were large, and spending on staff was the main cost component. The cost per neonatal life-year saved ranged from $135 to $1627. The intervention was highly cost-effective when using income-based thresholds. Variation in cost-effectiveness across trials was strongly correlated with costs. Removing discounting of costs and life-years substantially reduced all cost-effectiveness ratios. The cost of rolling out the intervention to rural populations ranges from 1.2% to 6.3% of government health expenditure in the four countries. Our analyses demonstrate the challenges faced by economic evaluations of community-based interventions evaluated using a cluster randomized controlled trial design. Our results confirm that women’s groups are a cost-effective and potentially affordable strategy for improving birth outcomes among rural populations.

**Key Messages**

- Despite a WHO recommendation and several randomized controlled trials, evidence gaps pose a significant barrier to the wider uptake of participatory learning and action with women’s groups, an effective strategy to reduce neonatal death.
- After reanalysing primary data and standardizing methods and assumptions, we find wide variation in cost and cost-effectiveness estimates persists.
- Spending on staff is by far the largest cost category. Understanding variation in unit costs is key to explaining differences in cost-effectiveness ratios; however, scale of delivery has limited value in explaining those differences.
- Scaling up to all rural areas in the four countries studied here (India, Nepal, Bangladesh and Malawi) is feasible and affordable.

## Introduction

An estimated 47% of the 5.4 million deaths of children under 5 years occur in neonates <28 days old (Hug *et al.*, 2018). Persistently high neonatal mortality rates at 18 deaths per 1000 live births globally and at 26 per 1000 live births in the least developed countries highlight the need to improve neonatal health outcomes through the wide-scale implementation of evidence-based, cost-effective interventions (Bhutta *et al.*, 2014; Hug *et al.*, 2018).

Exposure to community mobilization through facilitated participatory learning and action cycles with women’s groups (henceforth ‘women’s groups’) is associated with a significant and sizeable reduction in neonatal mortality across a range of settings (Manandhar *et al.*, 2004; Azad *et al.*, 2010; Tripathy *et al.*, 2010; Colbourn *et al.*, 2013a; Fottrell *et al.*, 2013; Lewycka *et al.*, 2013). A systematic review and a meta-analysis of effect estimates from seven randomized controlled trials found that exposure to groups was associated with a 33% reduction in neonatal mortality and a 49% reduction in maternal mortality when over 30% of pregnant women reported ever participating in a group (Prost *et al.*, 2013). The systematic review also assessed the cost-effectiveness evidence available from four of the trials at the time (Borghi *et al.*, 2005; Tripathy *et al.*, 2010; Fottrell *et al.*, 2013; Lewycka *et al.*, 2013), and, after basic adjustments for a common reporting year, concluded that women’s groups are a highly cost-effective intervention by World Health Organization (WHO) criteria (Tan-Torres Edejer *et al.*, 2003; Prost *et al.*, 2013). On the basis of these analyses, and the WHO’s own evidence review, women’s groups are now a recommended strategy for reducing maternal and neonatal mortality (World Health Organization, 2014).

The WHO recommendation does, however, necessitate a better understanding of the likely cost-effectiveness and the resources required to implement women’s groups at scale. The systematic review found wide variation in cost-effectiveness estimates from $91 to $753 per neonatal life-year saved (LYS) (in 2011 international $) from four of the trials (Prost *et al.*, 2013). Since then, two new estimates at the lower end of this range have been reported: $79 per LYS (in 2013 international $) from one of the trials included in the systematic review (Colbourn *et al.*, 2015), and $83 per LYS (in 2017 USD) from a trial that used community health workers incentivized under the National Health Mission of India (Sinha *et al.*, 2017). Cost-effectiveness estimates published in the individual trial papers have been used to compare women’s groups with similar interventions (Pitt *et al.*, 2016).

Current evidence is therefore an informative starting point but further comparative and in-depth analysis of the economic data from the original trials is warranted to inform prioritization processes and funding decisions (Wiseman *et al.*, 2016). Previously published costs of women’s groups reported only summary costs, without substantive detail. The main cost components of the intervention, the reasons for the wide variance in cost and cost-effectiveness estimates from the different trials, and the expected cost of intervening at scale, remain unknown. This article provides both substantive detail and commentary on the variation between costs across settings. Analyses of this sort have previously informed the evidence base around the use of community health workers in maternal and newborn care (Daviaud *et al.*, 2017). We used effectiveness estimates from the meta-analysis (Prost *et al.*, 2013), and sourced the primary cost data from six trials included in that analysis.

## Methods

### Overview of women’s group trials

The systematic review identified seven women's group trials in six locations across four countries: India, Nepal, Bangladesh and Malawi (Prost et al., 2013). All trials used a cluster randomized controlled design to evaluate the effectiveness of a community participatory learning and action cycle using women’s groups to reduce neonatal and maternal deaths. Although the content of the group discussions was targeted at women of reproductive age, groups were open to all women. All except Malawi-MaiKhanda implemented health service strengthening in both intervention and control areas, but otherwise the control clusters carried on with current practice. More detailed explanations of the intervention and trial characteristics can be found elsewhere: India (Tripathy *et al.*, 2010; More *et al.*, 2012), Nepal (Manandhar *et al.*, 2004), Bangladesh (Azad *et al.*, 2010; Fottrell *et al.*, 2013) and Malawi (Colbourn *et al.*, 2013a; Lewycka *et al.*, 2013).

Table 1 explains which of the seven trials have had cost-effectiveness analyses previously published, and which are included in this article. Sensitivity analyses have been conducted for the two trials that published separate cost-effectiveness reports (Borghi *et al.*, 2005; Colbourn *et al.*, 2015). Primary cost data were not collected for the one urban trial located in Mumbai, India (More *et al.*, 2012) and costs could not be estimated retrospectively without introducing significant bias. We therefore excluded it from this analysis.

**Table 1:** Summary of previously published cost-effectiveness evidence

Trial	Cost-effectiveness analysed in trial-specific paper	Cost-effectiveness included in systematic review (Prost *et al.*, 2013)	Cost data re-analysed in this paper
India (Ekjut)	Yes (Tripathy *et al.*, 2010)	Yes	Yes
India (Mumbai)	No	No	No
Nepal	Yes (Borghi *et al.*, 2005)	Yes	Yes
Bangladesh I	No	No	Yes
Bangladesh II	Yes (Fottrell *et al.*, 2013)	Yes	Yes
Malawi-MaiMwana	Yes (Lewycka *et al.*, 2013)	Yes	Yes
Malawi-MaiKhanda	Yes ( Colbourn *et al.*, 2015)	No	Yes

The target population was the same in all six trials: all pregnant women living in the study area. Despite a high degree of similarity in design and implementation sufficient to warrant meta-analysis of effectiveness, there were differences between the trials including: the duration, coverage and intensity of the intervention, and the size of the targeted population. These differences are described in Table 2. For example, in the first trial in Nepal, the intervention period was relatively short (24 months) and the intervention area population relatively small (86 704). Intervention coverage as defined in the systematic review (Prost et al., 2013) was relatively high, at 37% of pregnant women having attended at least one women’s group meeting. Intervention intensity, which can be measured using the number of women’s groups and average length of the intervention cycle, was relatively low (*n* = 111 groups; 10 meetings per group). By comparison, in the other trials, the intervention period was up to 12 months longer, and coverage ranged from 3% (Bangladesh I) to 51% (Malawi-MaiMwana); intensity was higher (highest in Bangladesh II at 810 groups and 24 meetings per group); and the target area population was larger (largest in Malawi-MaiKhanda at 1.2 million). The trials were of substantially different sizes: the Nepal study had c.3000 live births, compared with 100 000 in Malawi-MaiKhanda.

**Table 2:** Description and comparison of the interventions

Characteristic	India	Nepal	Bangladesh I	Bangladesh II	Malawi-MaiMwana	Malawi-MaiKhanda
Intervention period (months)	36	24	35	30	36	27
Cost-effectiveness time horizon (months)	49	48	66	42	55	48
Intervention area population	114 141	86 704	229 195	243 341	94 992^a^	1 200 000^a^^,b^
Live births^c^	9469	2899	15 153	8819	9174^a^	100 000^a^^,b^
Women’s groups	244	111	162	810	207^a^	729^a^
Meetings per group (average)	20	10	20	24	20	16
Intervention coverage (%)^d^	37	37	3	36	51	10

The figures are based on published papers (Manandhar *et al.*, 2004; Borghi *et al.*, 2005; Azad *et al.*, 2010; Tripathy *et al.*, 2010; More *et al.*, 2012; Colbourn *et al.*, 2013a,  2013b, 2015; Fottrell *et al.*, 2013; Lewycka *et al.*, 2013; Prost *et al.*, 2013).

a Full intervention area. Subsequently, we use figures relating to half this area (women’s groups only arm), as described in the Methods section.

b Estimated. Birth and death surveillance data were captured on an estimated 9% of total births.

c During the intervention period.

d Percentage of pregnant women who reported having attended at least one women’s group meeting (2013).

### Intervention effectiveness

The WHO recommendation (World Health Organization, 2014) focused on women’s groups to reduce neonatal mortality, as this was supported by the overall meta-analysis (Prost *et al.*, 2013). We therefore focus our analyses of cost-effectiveness on this outcome. We calculated LYS from the reduction in the neonatal mortality rate in each trial as reported in the meta-analysis (see Table 2B in Prost *et al.*, 2013). For Malawi-MaiKhanda, the number of recorded deaths was multiplied by 11 to adjust for the fact that only about 9% of the area was randomly selected to be under surveillance over the intervention periodColbourn *et al*., 2013b). Neonatal deaths averted were multiplied by 30.81 to generate a measure of LYS. This corresponds to assuming a standard life expectancy of 86 years, a 3% discount rate and no age weighting, as recommended in the 2010 Global Burden of Disease Study (Murray *et al.*, 2012; World Health Organization, 2017).

### Intervention cost

The original economic evaluations for these trials, which are described extensively elsewhere (Borghi *et al.*, 2005; Tripathy *et al.*, 2010; Fottrell *et al.*, 2013; Lewycka *et al.*, 2013; Colbourn *et al.*, 2015), prospectively collected cost data from a provider perspective and applied a step-down costing methodology. For the analyses presented here, we inputted the source cost data from the individual trials into a single, standardized Excel-based tool. Data categories and the procedure for allocating costs between cost centres were harmonized across trials, as we have previously described elsewhere (Batura *et al.*, 2014). The trial designs in Bangladesh and Malawi presented two specific costing challenges that had to be addressed to ensure comparability of estimates across countries. Supplementary Appendix S1 gives further details of how costs were identified in the original evaluations; the costing challenges specific to Bangladesh and Malawi; and the conversion of figures to 2016 international dollars (INT$, henceforth $).

### Analysing costs

We calculated total, annual and unit costs using the parameters shown in Table 2. Total cost of the women’s group intervention was computed as the sum of all start-up and implementation costs over the time horizon used for each trial’s cost-effectiveness analysis. This was consistent with the original evaluations, which conservatively included the costs of all activities during the start-up period (excluding research activities), such as staff recruitment and training, securing community approval and adapting intervention delivery methods, content and materials to the local context. A share of recurrent costs during the implementation period was also included as start-up costs, to reflect the recruitment and training of replacement staff. Total cost was divided by the cost-effectiveness time horizon to compute annual total cost. Implementation cost was divided by the intervention period to compute annual implementation cost.

We calculated three different unit cost estimates with reference to population size and the number of women’s groups in the intervention area: cost per live birth, annual cost per person and annual cost per group. Cost per live birth was computed by dividing total cost by the number of live births during the intervention period, which represents the population of potential beneficiaries of the intervention in relation to the main outcome measure, neonatal deaths averted. Annual cost per person and annual cost per group were computed by dividing annual total cost by the total population (all ages) living in the intervention area and the number of women’s groups, respectively. The design of the trials and the characteristics of the women’s group intervention precluded the identification and measurement of resource use on the individual level, and thus the estimation of unit costs at the level of individual intervention participants (Batura *et al.*, 2014).

We explored the components of total cost by computing the proportion of total costs for each of the four data categories: staff (including programme staff, women’s group facilitators and supervisors), materials, other recurrent (items such as transportation, communication, utilities, bank charges, etc.) and capital costs. A more detailed break-down was not possible due to differences in the level of detail in the primary cost data. In particular, due to lack of disaggregated data on staff costs from all six trials, we were not able to examine variation in factors such as the number of staff involved in intervention implementation, their remuneration levels and staff productivity.

### Cost-effectiveness

The cost-effectiveness ratio was calculated in the base case as cost per neonatal LYS. We compared the estimates with income-based thresholds that have been recommended by WHO, which suggest in our case that the intervention is ‘very cost-effective’ if the cost per LYS is less than annual gross domestic product (GDP) per capita, and ‘cost-effective’ if it is less than three times per capita GDP (Commission on Macroeconomics and Health, 2001). These thresholds have since come under criticism, and alternative methods for estimating thresholds have been developed (e.g. Bertram *et al.*, 2016; Culyer, 2016; Woods *et al.*, 2016). We used the WHO-recommended thresholds because they are currently the most widely applied. However, we also discuss the implications of a lower threshold.

The analytical methods and reporting of the cost-effectiveness results follow the Consolidated Health Economic Evaluation Reporting Standards Statement (Husereau *et al.*, 2013). The completed checklist is provided in Supplementary Appendix S2.

### Exploring reasons for variation

We explored the possible reasons for variation in cost-effectiveness ratios across countries using simple two-way scatter plots and the Pearson’s correlation coefficient. First, we examined whether cost per neonatal LYS was more strongly associated with effectiveness (the number of LYS) or with unit costs (cost per live birth). Second, we compared unit costs and the cost-effectiveness ratio with coverage, scale and intensity of the intervention. Coverage, defined as the proportion of pregnant women who report having attended at least one women’s group meeting, was previously found to be a significant determinant of effectiveness (Prost *et al.*, 2013). Scale was measured by the number of live births and the total intervention area population. Intensity was measured by the number of women’s groups. A *P*-value of <0.05 was used to determine significance.

### Cost, affordability and outcomes of national scale-up

The cost, affordability and outcomes of national scale-up in Bangladesh, India, Malawi and Nepal were then estimated to inform national policy. Previously, the affordability of national delivery has been examined only for Malawi (Colbourn *et al.*, 2015). Scale-up analyses assumed delivery of the intervention to the whole rural population, over a 1-year period. Cost was estimated using the average annual cost per person from the trial for that context. Since our own analyses found no conclusive evidence of economies of scale (see Results section), we assumed that cost per person is constant when the intervention is scaled-up. The benefits of intervening at scale were estimated, taking the same approach as in the meta-analysis (Prost *et al.*, 2013), but updating the population parameters with more recent values. As the effectiveness of a trial may not be maintained at scale (Hanson *et al.*, 2015), we provide two estimates of effect at scale, an upper and a lower bound. For the upper bound, we assumed that the scaled-up intervention will have the same effectiveness as reported in the meta-analysis of high coverage trials i.e. a 33% reduction in neonatal mortality. To estimate a lower bound, we assumed a 30% loss of effectiveness when the intervention is implemented at scale. Supplementary Appendix S3 summarizes the population data used for these calculations and describes the methods in more detail.

### Sensitivity analysis

The base case is the ‘best’ estimate of cost-effectiveness, measured with prospective cost and effect data. It is against this base case that the sensitivity of cost-effectiveness to changes in the assumptions and estimated parameters was formally compared using deterministic one-way sensitivity analysis.

We first added maternal LYS to the estimated neonatal LYS to explore the resulting effect on the cost-effectiveness ratio. Maternal mortality was not included in our base case because of the lack of statistical significance in the overall meta-analysis (odds ratio 0.77, 95% confidence interval 0.48–1.23). However, limiting the base case to neonatal LYS represents a highly conservative estimate of the health effects of women’s groups. The meta-analysis found that in the four trials where at least 30% of women had attended women’s groups, the intervention had a significant effect on maternal mortality (Prost *et al.*, 2013). We therefore used the adjusted odds ratio for maternal mortality in each trial (Prost *et al.*, 2013), and multiplied the number of maternal deaths averted by the life expectancy that corresponds to the average age at death in each trial (between 26 and 30), to calculate maternal LYS. A 3% discount rate was applied. The meta-analysis also examined effects on stillbirths but found no evidence of a reduction. We therefore did not consider LYS from stillbirths.

Second, we reduced the start-up costs of all trials by 50%. This reflects the assumption that while all trials had a relatively long start-up period (as is typical of community interventions), once an intervention has been tested in a context and standardized, it is very likely that the start-up period and associated costs would reduce significantly.

Third, we varied the trial-specific joint cost allocation rules that were used in the original economic evaluations. The joint cost allocation rule decides which percentage of common (shared) staff, material, capital and other recurrent costs, should be allocated to the women’s group intervention as opposed to other activities, such as monitoring and evaluation, process evaluation, other interventions or research. We varied the allocated share up and down, by 10 percentage points from the original allocation.

Fourth, we conducted a specific sensitivity analysis for the two Malawi trials that tested another intervention alongside women’s groups (see Supplementary Appendix S1 for details). The proportion of women’s group implementation costs allocated to the women’s groups only arm was varied between a 33% lower bound and a 75% upper bound. This can be interpreted as reflecting alternative scenarios regarding economies of scale and scope when two interventions are implemented in the same trial.

Finally, we explored two alternatives to the 3% discount rate for both costs and outcomes (NICE International, 2014): a 0% rate for both costs and life years, and a differential scenario of 6% for costs and 3% for life years (Claxton *et al.*, 2011).

### Role of the funding source

The funder of the study had no role in study design, data collection, data analysis, data interpretation or writing of the report. The corresponding author had full access to all the data in the study and had final responsibility for the decision to submit for publication.

## Results

### Differences in resource use

Table 3 presents a summary of the standardized base-case costs. The total cost of the women’s group intervention varied from about $800 000 (India) to $3 million (Malawi-MaiKhanda). The average annual cost was $406 555 (Median: $320 680). Breaking this down into start-up and implementation costs, we found that total start-up costs were substantial in all trials, averaging at $551 541 (median: $481 329) or 33% (range: 15–51%) of total costs. Large variations in annual implementation costs were observed, with 6-fold variation around the mean value of $271 730 (median: $203 124).

**Table 3:** Cost description of the women’s groups intervention (2016 INT$)

	India	Nepal	Bangladesh I	Bangladesh II Modelled	Malawi-MaiMwana^a^	Malawi-MaiKhanda^a^	Mean
Total cost	797 212	1 556 020	1 387 949	2 237 115	904 504	3 064 842	1 657 941
Start-up cost	308 810	517 970	711 312	1 094 497	231 969	444 687	551 541
Annual total cost^b^	195 236	389 005	252 354	639 176	197 346	766 211	406 555
Annual cost of implementation^c^	119 609	259 512	123 025	326 462	146 735	655 039	271 730

a Women’s groups only arm (see Methods section).

b Averaged over the cost-effectiveness time horizon (see Table 2).

c Averaged over the intervention period (see Table 2).

Decomposition of the total costs into unit costs of delivery is presented in Table 4. The average cost per live birth was $203 (median: $142). Variation across countries was large: the cost in Nepal ($537 per live birth) was over eight times the cost in Malawi-MaiKhanda ($61 per live birth). There was a 4.5-fold difference in the annual cost of facilitating a group, ranging from $800 per group in India, to $3505 per group in Nepal. A similar difference was observed for the annual cost per person.

**Table 4:** Unit costs of the intervention (2016 INT$)

Unit costs	India	Nepal	Bangladesh I	Bangladesh II Modelled	Malawi-MaiMwana	Malawi-MaiKhanda	Mean
Cost per live birth	84	537	92	254	193	61	203
Annual cost per group	800	3505	1558	789	1907	2102	1777
Annual cost per person (all ages)	1.7	4.5	1.1	2.6	4.2	1.3	2.6

**Figure 1:**
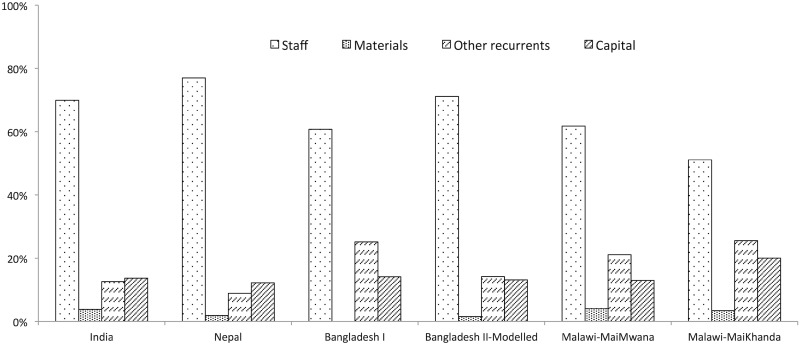
Components of total cost.

While the total, annual and unit costs of the women’s group intervention varied significantly across countries, Figure 1 illustrates that the composition of those costs did not vary to the same extent. Staff costs, and to a lesser extent transport costs, were the only substantial variable costs within total trial spending. Staff costs accounted on average for 65% of total spending; ranging from 51% in Malawi-MaiKhanda to 77% in Nepal (Figure 1). Material costs, on average, comprised only 2% of total intervention costs. The ‘other recurrents’ category constituted an average of 18% of intervention costs. This proportion differed substantially across countries from 9% in Nepal to 26% in Malawi-MaiKhanda. Capital costs constituted a similar proportion of total costs at an average of 14%, with the exception of Malawi-MaiKhanda, where capital costs comprised 20% of the total intervention cost. Supplementary Appendix S4 reports the annual aggregate costs by cost component for each trial.

### Cost-effectiveness

The effectiveness of the women’s group intervention has been extensively described elsewhere (Prost *et al.*, 2013). In short, an estimated 782 neonatal deaths were averted by the intervention across the four countries. This varied from 31 cases in Nepal to 350 in Malawi-MaiKhanda (Table 5). The total (discounted) neonatal LYS was estimated at 24 085 or an average of 4012 LYS per trial.

**Table 5:** Effectiveness and cost-effectiveness of women’s groups (2016 INT$)

	India	Nepal	Bangladesh I	Bangladesh II Modelled	Malawi-MaiMwana	Malawi-MaiKhanda	Mean
Neonatal deaths averted	191	31	57	115	38	350	130
Neonatal LYS^a^	5887	956	1763	3531	1178	10 770	4014
Cost per neonatal LYS	$135	$1627	$787	$634	$768	$285	$706
GDP per capita, PPP for 2016^b^	$6572	$2468	$3581	$3581	$1169	$1169	N/A

a Discounted at 3%.

b Threshold value for ‘very cost-effective’ interventions.

Cost-effectiveness ratios varied substantially between the countries from $135 per neonatal LYS in India to $1627 per neonatal LYS in Nepal (Table 5). Despite these considerable differences, the women’s group intervention was a highly cost-effective intervention in each country when using the income-based thresholds recommended by WHO. Supplementary Appendix S5 summarizes how these results compare with previously published cost-effectiveness ratios.

### Reasons for differences in cost and cost-effectiveness ratios

**Figure 2:**
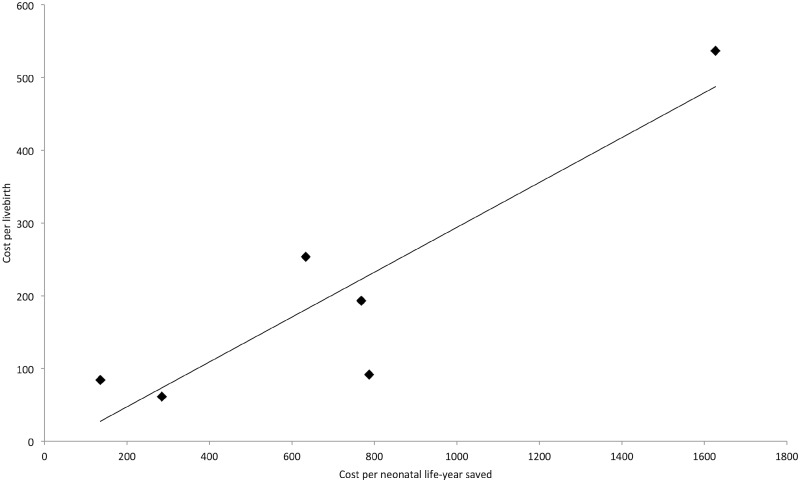
Association between the cost-effectiveness ratio and unit costs.

Using the six data points provided by the six trials, we explored possible reasons for variation in cost-effectiveness ratios across countries. The only statistically significant association that was found was between unit costs and the cost-effectiveness ratio (*r* = 0.90, *P* = 0.01; Figure 2). That is, trials with a low cost per live birth tended to have a smaller cost per LYS. Effectiveness (in terms of the number of LYS) was less strongly associated with the cost-effectiveness ratio (*r* = −0.71, *P* = 0.11).

Our data suggested that differences in intervention coverage do not explain differences in cost (*r* = 0.45, *P* = 0.37) and, even less so, differences in cost-effectiveness ratios (*r* = 0.20, *P* = 0.71). While there was some suggestion of positive economies of scale for potential beneficiaries (i.e. a negative correlation between the number of new-borns and cost per live birth; *r* = −0.54, *P* = 0.26); this evidence of a relationship between scale and unit costs was too weak to be used in the scale-up modelling. We therefore subsequently assumed that cost per person is constant when the intervention is scaled-up. Intervention intensity, indicated by the number of women’s groups, was not associated with either cost per live birth (*r* = −0.09; *P* = 0.86) or the cost-effectiveness ratio (*r* = −0.32; *P* = 0.53). Full results of these analyses are presented in Supplementary Appendix S6.

The study from Nepal stands out in these analyses. This is where the intervention was delivered at the smallest scale but with high coverage. It had both the highest unit cost ($537 per life birth) and the highest cost-effectiveness ratio ($1627 per LYS). These features fit well into the overall pattern that emerges and are perhaps unsurprising given that Nepal was the first trial to evaluate the women’s group intervention. The costs of scaling up to national delivery will be explored next.

### Cost, affordability and outcomes of scaling up the intervention

Table 6 presents the cost and affordability of the women’s group intervention when scaled-up to rural areas of India, Nepal, Bangladesh and Malawi. We estimate that the annual cost of scaling up the intervention to cover the whole rural population is $1514 million in India, $105 million in Nepal, $278 million in Bangladesh and $41 million in Malawi (in 2016 INT$). The average annual scale-up cost is 2.0% of total national health expenditure, 4% of government health expenditure and 0.1% of national GDP, across all settings. There is considerable variation in affordability estimates; the cost ranges from 0.37% of total national health expenditure in India to 2.54% in Malawi. In 2016, the contributions of health expenditure to GDP were 4.7% in India, 5.8% in Nepal, 2.8% in Bangladesh and 11.4% in Malawi. The intervention would represent 1.2% of government health expenditure in India, 6.3% in Nepal, 6.1% in Bangladesh and 3.2% in Malawi. Scaling up to e.g. 50% of the rural population would imply cost and affordability equal to half the figures presented in Table 6.

**Table 6:** Cost and affordability of scaling up to national delivery

Description	India	Nepal	Bangladesh^a^	Malawi^b^
Average annual cost (million $)	1514	105	278	41
% Total health expenditure	0.37	2.54	1.70	1.70
% Government health expenditure	1.23	6.30	6.10	3.23
% GDP	0.02	0.15	0.05	0.19

a Data and assumptions for Bangladesh II Modelled were used here.

b Mean value of unit costs for Malawi-MaiMwana and Malawi-MaiKhanda was used here.

To put these costs into perspective, we updated the expected outcome estimates from Prost *et al.* (2013) and present the results in terms of neonatal lives saved in Table 7. Scaling up the intervention to the whole rural population could prevent around 61 000 neonatal deaths, around 9% of the total burden in the four countries under study. This ranges from 3% in Malawi to 15% in Nepal (Table 7). The greatest number of neonatal lives would be saved in India, with around 43 000 lives. When effectiveness is assumed to reduce by 30% as a result of the increased scale of delivery, the reduction in neonatal deaths is 7% on average (range: 2–11%).

**Table 7:** Estimated number of neonatal lives saved by scaling up women’s groups

Countries	Assuming NO loss of effectiveness at scale		Assuming 30% loss of effectiveness at scale	
	No. neonatal lives saved	% of total neonatal deaths	No. neonatal lives saved	% of total neonatal deaths
India	42 780	5%	29 946	3%
Nepal	2527	15%	1769	11%
Bangladesh	15 445	14%	10 812	10%
Malawi	473	3%	331	2%
**Total**	61 226	9%	42 858	7%

### Sensitivity analysis

Table 8 summarizes the sensitivity of the base-case cost-effectiveness results to changes in the assumptions and estimated parameters. Adding maternal LYS to the neonatal LYS, reduces the cost-effectiveness ratio by 19% in Nepal and 25% in Malawi-MaiMwana and has a modest effect (between 4% and 9% reduction) on the cost per LYS in other trials. Reducing the start-up costs from 100% to 50% reduces the cost per (neonatal) LYS by 18% on average (around 25% in Bangladesh). Varying the joint cost allocation rule (by 10 percentage points in either direction) has a modest impact on the results (between 3% and 15%).

**Table 8:** Results of one-way sensitivity analyses on cost per LYS (2016 INT$)

Scenarios/ Parameters	India	Nepal	Bangladesh I	Bangladesh II Modelled	Malawi-MaiMwana	Malawi-MaiKhanda
Base-case scenario	135	1627	787	634	768	285
GDP per capita	6572	2468	3581	3581	1169	1169
Health outcomes (Base-case neonatal LYS only)						
Add maternal LYS^a^	123	1325	N/A	610	576	268
Start-up costs (Base case 100%)						
Reduce start-up costs by 50%	109	1356	586	479	670	264
Joint cost allocation rules^b^						
Base-case allocation rule (%)	29–36%	N/A	40%	40%	25%	30–35%
−10% points	126	N/A	713	614	689	242
+10% points	145	N/A	862	653	847	328
Inclusion of implementation costs in factorial trials (Base case 50%)						
33% costs included	N/A	N/A	N/A	N/A	574	202
75% costs included	N/A	N/A	N/A	N/A	1054	406
Discount rate (Base case 3% both costs and life years)						
Costs 0%, life years 0%	52	623	302	234	295	106
Costs 6%, life years 3%	127	1526	737	616	720	276

a Discounted at 3%.

b It was not possible to run this analysis for Nepal.

Standardizing costs across the two trials with a factorial design (Malawi-MaiKhanda and Malawi-MaiMwana) implied here that we estimated cost and effect in the women’s groups only arm in both trials (see Supplementary Appendix S1 for details). The cost-effectiveness ratio is sensitive to changing the assumption that 50% of women’s group implementation costs occurred in this arm. Including only 33% of implementation costs, on average, decreases the cost-effectiveness ratio by 27%, while including 75% of the costs, increases the ratio by 40%. Conclusions regarding cost-effectiveness are not affected, however.

Finally, removing discounting of costs and life years reduces the cost-effectiveness ratio substantially (by 62–63%) for all trials. The differential scenario of a 6% discount rate for costs and 3% for life years has a modest impact (between 3% and 6% reduction) on the results. Overall, varying the discount rate does not change conclusions regarding cost-effectiveness of the intervention when using the income-based thresholds.

## Discussion

Comparing cost and cost-effectiveness across trials and interventions can be challenging. The complexity of this comparison is seldom explicitly explored in the literature. This article both engages with a formal process of comparing costs between contexts and between six trials of a similar intervention, exploring and enacting the adjustments needed for direct comparison. In addition, this article makes an important empirical contribution to our understanding of the cost-effectiveness of women’s groups and the determinants of cost variation across contexts. It also expands the evidence regarding the affordability of national delivery, previously examined for Malawi (Colbourn *et al.*, 2015), to India, Nepal and Bangladesh.

The findings describe large differences in unit costs ($61–$537 per live birth) as well as in the scale and intensity of the intervention. After harmonizing methods and assumptions, cost-effectiveness ratios still vary widely from $135 to $1627 per neonatal LYS yet fall well below income-based cost-effectiveness thresholds. Scaling up the intervention to rural populations is expected to cost 6.3% of government health expenditure in Nepal, 6.1% in Bangladesh, 3.2% in Malawi and 1.2% in India.

The cost profile of the women’s group intervention is similar to that of newborn home visits (Pitt *et al.*, 2016). Staff costs constitute by far the largest proportion of total costs: on average 65% for women’s groups and 75% for home visits. Somewhat surprisingly for community-based interventions, capital costs are also substantial, at an average of 14% for women’s groups and 15% for home visits. This suggests scale-up plans should take care to budget for and invest in capital items, in particular vehicles, and IT and office equipment, which facilitate effective supervision.

None of the three factors explored here (intervention coverage, scale and intensity) significantly explained differences in unit costs and cost-effectiveness ratios. The type of staff used as facilitators, and their remuneration levels, emerges as an important topic for further study. While our analyses were not powered to compare modes of delivery, results from Malawi-MaiKhanda suggest a ‘volunteer-based’ model delivered on a large scale can have a relatively low unit cost and be highly cost-effective. Another trial in India found that using community health workers (ASHAs) was equally effective as separately recruited facilitators, but somewhat more costly and less cost-effective (Sinha *et al.*, 2017). Nevertheless, the unit cost ($124 per live birth in 2016 INT$) and cost-effectiveness ratio ($295 per neonatal LYS) from that trial compare favourably with the other countries included here. Further evidence on the impact on cost and cost-effectiveness is likely to emerge as scale-up of ASHA-facilitated women’s groups proceeds in India.

Our study has three main limitations. First, women’s groups are likely to have benefits for the mother and child, which are not reflected in the cost-effectiveness ratio. These may include lower morbidity, long-term health benefits, health benefits for siblings, as well as non-health benefits such as loan availability, consumption smoothing and environmental benefits. In our sensitivity analysis, we incorporate maternal LYS, and find the cost-effectiveness ratios in Nepal and Malawi-MaiMwana reduce by 19% and 25%, respectively. However, the full effects on mortality in any individual trial, or the broader benefits of the intervention, are not captured here (Prost *et al.*, 2013).

Second, our estimates use costs observed in a trial setting from a provider perspective. The fact that the original trial costings mainly sourced data from expenditure reports and financial records, may imply that our scale-up cost estimates are overstated (Cunnama *et al.*, 2016). On the other hand, estimation of costs from the societal perspective would require the inclusion of costs (in particular time use) incurred by women and other community members. Previously, we have discussed the inherent difficulties in disentangling intervention costs from research costs and the allocation of joint (shared) costs in these trials (Batura *et al.*, 2014). Outside a trial setting, joint costs may be smaller, and the start-up period may be shorter. These two aspects are addressed in our sensitivity analysis. However, we were unable to perform formal sensitivity analysis on the impact on cost of the involvement of expatriate or overseas staff in the start-up and/or implementation stages of the intervention in each country. While using local staff will likely imply a lower implementation cost, it may also impact on intervention effectiveness (Colbourn *et al.*, 2015).

Third, we have used the WHO threshold for cost-effectiveness in our study (Commission on Macroeconomics and Health, 2001). However, we acknowledge the recent discussions on alternative supply-side cost-effectiveness thresholds which are much lower than the WHO-recommended threshold, possibly <60% GDP per capita (Woods *et al.*, 2016; Ochalek *et al.*, 2018). Using this threshold, the intervention is potentially cost-effective in India (2% GDP per capita), Malawi-MaiKhanda (24% GDP per capita), Bangladesh I (22% GDP per capita) and Bangladesh II Modelled (18% GDP per capita), but not in Nepal, or in Malawi-MaiMwana (both 66% GDP per capita).

## Conclusion

Our findings support previous conclusions that large-scale implementation of women’s groups is a cost-effective and potentially affordable strategy that could save around 43 000 neonatal lives each year in the four countries studied here. Evidence of cost-effectiveness can provide decision-makers with information on how to allocate resources between competing priorities, but this must be accompanied by other considerations. In the case of women’s groups, these might include the opportunity cost in relation to other available health or social interventions; the equity implications of women’s groups; community willingness to keep the groups running; strategies to improve access to antenatal and delivery services; and working with groups to improve health and non-health outcomes beyond the perinatal period (Drummond *et al.*, 2005; Yukich *et al.*, 2008; Conteh *et al.*, 2010; Houweling *et al.*, 2013). Should national agencies proceed with the implementation of women’s groups to improve maternal and neonatal mortality and morbidity, they are urged to consider the coverage of those groups, and the levels of staffing required to achieve and maintain that coverage.

## Supplementary data

Supplementary data are available at *Health Policy and Planning* online

## Supplementary Material

czaa081_Supplementary_Data
