# Participatory learning and action cycles with women's groups to prevent neonatal death in low-resource settings: A multi-country comparison of cost-effectiveness and affordability

**DOI:** 10.1093/heapol/czaa164

**Published:** 2020-12-29

**Authors:** Anni-Maria Pulkki-Brännström, Hassan Haghparast-Bidgoli, Neha Batura, Tim Colbourn, Kishwar Azad, Florida Banda, Lumbani Banda, Josephine Borghi, Edward Fottrell, Sungwook Kim, Charles Makwenda, Amit Kumar Ojha, Audrey Prost, Mikey Rosato, Sanjit Kumer Shaha, Rajesh Sinha, Anthony Costello, Jolene Skordis

*Health Policy and Planning*, October 2020, https://doi.org/10.1093/heapol/czaa081 Published: 21 October 2020

In the originally published version of this manuscript, several errors were noted and listed in this corrigendum.

Upon the original publication, there was an error in the title. It should read: “Participatory learning and action cycles with women’s groups to prevent neonatal death in low-resource settings: A multi-country comparison of cost-effectiveness and affordability”.

Upon the original publication, the results presented in Supplementary Appendix S6 was omitted from the Supplementary data.

Upon the original publication, the Acknowledgments omitted the following statement: “We thank Bidur Thapa of MIRA, Nepal who contributed to the acquisition of data for the work.”

These have now been corrected online.

