# Supplementary material for: Participatory learning and action cycles with women’s groups to prevent neonatal death in low-resource settings: A multi-country comparison of cost-effectiveness and affordability
Source: Health Policy Plan. 2020 Oct 21;35(10):1280–9. doi: 10.1093/heapol/czaa081 (PMC7886438; doi:10.1093/heapol/czaa081)
Supplement: czaa081_Supplementary_Data [file czaa081_supplementary_data.zip › Appendix 1 Identifying and standardising costs.docx]

## Appendix 1: Identifying and standardising costs

The original economic evaluations ([Tripathy et al., 2010](#_ENREF_10), [Fottrell et al., 2013](#_ENREF_6), [Lewycka et al., 2013](#_ENREF_7), [Borghi et al., 2005](#_ENREF_3), [Colbourn et al., 2015](#_ENREF_5)) applied a step-down costing methodology, whereby costs from each site’s project accounts were input into an MS Excel sheet designed for the purpose. Information on staff time-use and the timing of programme activities were used qualitatively to inform decisions on the allocation of costs spanning multiple trial activities (e.g. monitoring and evaluation), and joint costs. The purchase price of capital items was annualised over their expected lifetime. Data input and cost allocation decisions were made by local finance and accounting staff at each location, supported and supervised by researchers.

The design of the trials and the characteristics of the women’s group intervention preclude the estimation of unit costs at the level of individual intervention participants (20). The women’s group intervention is delivered at the community level and thus individual exposure is not observable. In the trials however, intervention effects (health outcomes) were identifiable and measured at the individual level. The inability to identify and measure resource use on the individual level is a common limitation to the cost-effectiveness analyses of complex global public health interventions (20).

For the analyses presented here, we inputted the source cost data from the individual trials into a single, standardised Excel-based tool. Data categories and the procedure for allocating costs between cost centres was harmonised across trials, as we have previously described elsewhere ([Batura et al., 2014](#_ENREF_2)). We inflated yearly costs to 2016 values using local consumer price indices, and converted from local currency to international dollars using the 2016 purchasing-power parity (PPP) conversion factors for each country ([The World Bank](#_ENREF_9)). The exchange rates used were 1 INT$ = 29.69 Taka (Bangladesh), 17.45 INR (India), 183.63 Kwacha (Malawi), and 31.4 NPR (Nepal).

The trial designs in Bangladesh and Malawi presented two specific costing challenges that had to be addressed to ensure comparability of estimates across countries. For Bangladesh II and Malawi-MaiMwana, our base case followed the approaches outlined in the original papers ([Fottrell et al., 2013](#_ENREF_6), [Lewycka et al., 2013](#_ENREF_7)), which are briefly described below. For Malawi-MaiKhanda, we adopted a different approach in the base case, for reasons described below, which resulted in somewhat higher unit cost estimates than in the original analysis ([Colbourn et al., 2015](#_ENREF_5)).

For Bangladesh II, a challenge was to avoid underestimating the potential effect of activities during Bangladesh I, in which the same intervention was implemented in the same area, but with fewer women’s groups (162 vs. 810 groups; see Table 2 in the main manuscript). No effect on neonatal mortality was observed in Bangladesh I ([Azad et al., 2010](#_ENREF_1)), but a significant effect was found in Bangladesh II ([Fottrell et al., 2013](#_ENREF_6)). The original Bangladesh II cost-effectiveness analysis addressed this by presenting two estimates: a prospective cost that summed up the cost of both trials; and a “modelled” cost that included start-up cost of both trials and 2.5 years of implementation at the average annual implementation cost observed in Bangladesh II ([Fottrell et al., 2013](#_ENREF_6)). We adopted this latter approach, referred to hence forth as Bangladesh II-Modelled, and explored the sensitivity of the results to the inclusion of the full start-up costs in sensitivity analysis.

The challenge in the Malawi trials was related to the two-by-two factorial design, whereby women’s groups were implemented in two trial arms. In one of these, a second intervention was simultaneously implemented: an infant feeding peer counselling intervention in MaiMwana ([Lewycka et al., 2013](#_ENREF_7)), and a health facility quality improvement intervention in MaiKhanda ([Colbourn et al., 2013](#_ENREF_4)). In the base case, we assumed no economies of scale or scope between women’s groups and these other, potentially complementary interventions. We therefore estimated the cost of the “women’s group only” arm by adding up the full start-up cost of the women’s group intervention, and half (50%) of the women’s group implementation costs. This was also the approach used in the original Malawi-MaiMwana analysis ([Lewycka et al., 2013](#_ENREF_7)). In contrast, the original Malawi-MaiKhanda cost-effectiveness analysis used the total women’s group cost ([Colbourn et al., 2015](#_ENREF_5)). The main reason for this difference was that in MaiMwana, a significant interaction effect between the two interventions was found ([Lewycka et al., 2013](#_ENREF_7), [Prost et al., 2013](#_ENREF_8)). Thus, the effect size estimates are from a stratified analysis (comparison of women’s group only arm with control) in MaiMwana and from a factorial analysis in MaiKhanda.

We formally tested the robustness of our results to the base-case assumption assumption that in the Malawi trials, 50% of the women’s group implementation costs occurred in the women’s group only arm. We varied the allocation of implementation costs between a 33% lower bound and a 75% upper bound. The upper bound is consistent with economies of scale or scope (implementation cost increases less than proportionally) and the lower bound is consistent with diseconomies of scale or scope (implementation cost increases more than proportionally).

**References**

AZAD, K., BARNETT, S., BANERJEE, B., SHAHA, S., KHAN, K., REGO, A. R., BARUA, S., FLATMAN, D., PAGEL, C., PROST, A., ELLIS, M. & COSTELLO, A. 2010. Effect of scaling up women's groups on birth outcomes in three rural districts in Bangladesh: a cluster-randomised controlled trial. *The Lancet,* 375**,** 1193-1202.

BATURA, N., PULKKI-BRÄNNSTRÖM, A.-M., AGARWAL, P., BAGRA, A., HAGHPARAST-BIDGOLI, H., BOZZANI, F., COLBOURN, T., GRECO, G., HOSSAIN, T., SINHA, R., THAPA, B. & SKORDIS-WORRALL, J. 2014. Collecting and analysing cost data for complex public health trials: reflections on practice. *Global Health Action,* 7**,** 23257.

BORGHI, J., THAPA, B. & OSRIN, D. 2005. Economic assessment of a women's group intervention to improve birth outcomes in rural Nepal. *The Lancet,* 366**,** 1882-1884.

COLBOURN, T., NAMBIAR, B., BONDO, A., MAKWENDA, C., TSETEKANI, E., MAKONDA-RIDLEY, A., MSUKWA, M., BARKER, P., KOTAGAL, U., WILLIAMS, C., DAVIES, R., WEBB, D., FLATMAN, D., LEWYCKA, S., ROSATO, M., KACHALE, F., MWANSAMBO, C. & COSTELLO, A. 2013. Effects of quality improvement in health facilities and community mobilization through women's groups on maternal, neonatal and perinatal mortality in three districts of Malawi: MaiKhanda, a cluster randomized controlled effectiveness trial. *International Health,* 5**,** 180-195.

COLBOURN, T., PULKKI-BRÄNNSTRÖM, A.-M., NAMBIAR, B., KIM, S., BONDO, A., BANDA, L., MAKWENDA, C., BATURA, N., HAGHPARAST-BIDGOLI, H., HUNTER, R., COSTELLO, A., BAIO, G. & SKORDIS-WORRALL, J. 2015. Cost-effectiveness and affordability of community mobilisation through women's groups and quality improvement in health facilities (MaiKhanda trial) in Malawi. *Cost Effectiveness and Resource Allocation,* 13.

FOTTRELL, E., AZAD, K., KUDDUS, A., YOUNES, L., SHAH, S., NAHAR, T., AUMON, B. H., HOSSEN, M., BEARD, J., HOSSAIN, T., PULKKI-BRANNSTROM, A.-M., SKORDIS-WORRALL, J., PROST, A., COSTELLO, A. & HOUWELING, T. A. J. 2013. The effect of increased coverage of participatory women's groups on neonatal mortality in Bangladesh: A cluster randomized trial. *JAMA Pediatrics,* 167**,** 816-825.

LEWYCKA, S., MWANSAMBO, C., ROSATO, M., KAZEMBE, P., PHIRI, T., MGANGA, A., CHAPOTA, H., MALAMBA, F., KAINJA, E., NEWELL, M.-L., GRECO, G., PULKKI-BRÄNNSTRÖM, A.-M., SKORDIS-WORRALL, J., VERGNANO, S., OSRIN, D. & COSTELLO, A. 2013. Effect of women's groups and volunteer peer counselling on rates of mortality, morbidity, and health behaviours in mothers and children in rural Malawi (MaiMwana): a factorial, cluster-randomised controlled trial. *The Lancet,* 381**,** 1721-1735.

PROST, A., COLBOURN, T., SEWARD, N., AZAD, K., COOMARASAMY, A., COPAS, A., HOUWELING, T. A. J., FOTTRELL, E., KUDDUS, A., LEWYCKA, S., MACARTHUR, C., MANANDHAR, D., MORRISON, J., MWANSAMBO, C., NAIR, N., NAMBIAR, B., OSRIN, D., PAGEL, C., PHIRI, T., PULKKI-BRÄNNSTRÖM, A.-M., ROSATO, M., SKORDIS-WORRALL, J., SAVILLE, N., MORE, N. S., SHRESTHA, B., TRIPATHY, P., WILSON, A. & COSTELLO, A. 2013. Women's groups practising participatory learning and action to improve maternal and newborn health in low-resource settings: a systematic review and meta-analysis. *The Lancet,* 381**,** 1736-1746.

THE WORLD BANK [Internet]. World Development Indicators. Cited: September 2017: Available from: https://data.worldbank.org/indicator/PA.NUS.PPP.

TRIPATHY, P., NAIR, N., BARNETT, S., MAHAPATRA, R., BORGHI, J., RATH, S., RATH, S., GOPE, R., MAHTO, D., SINHA, R., LAKSHMINARAYANA, R., PATEL, V., PAGEL, C., PROST, A. & COSTELLO, A. 2010. Effect of a participatory intervention with women's groups on birth outcomes and maternal depression in Jharkhand and Orissa, India: a cluster-randomised controlled trial. *The Lancet,* 375**,** 1182-1192.
