# Supplementary material for: Participatory learning and action cycles with women’s groups to prevent neonatal death in low-resource settings: A multi-country comparison of cost-effectiveness and affordability
Source: Health Policy Plan. 2020 Oct 21;35(10):1280–9. doi: 10.1093/heapol/czaa081 (PMC7886438; doi:10.1093/heapol/czaa081)
Supplement: czaa081_Supplementary_Data [file czaa081_supplementary_data.zip › Appendix 3 Methods used to estimate cost and outcomes of national scale-up.docx]

## Appendix 3: Methods used to estimate cost and outcomes of national scale-up

Scale-up analyses assumed delivery of the intervention to the whole rural population, over a one-year period. Since our own analyses found no statistically significant evidence of economies of scale (see Appendix 3), we assumed that the average annual cost per person in each country was the same as in the trial for that context. Scale-up costs were therefore calculated by multiplying average annual cost per person (all ages) by the total rural population in each country. For Bangladesh we used Bangladesh II–Modelled, and for Malawi we used a simple average of the MaiMwana and MaiKhanda estimates.

The number of lives saved in one year of delivery at scale was estimated by:

*Bd_r_(1-RR)(1-s)*

where B is the number of rural births, dr is the neonatal mortality rate in rural areas, RR is the Risk Ratio for the intervention in rural deliveries for neonatal mortality; and s is the rate of skilled attendance in rural regions. To estimate an upper bound of likely effectiveness at scale, we assumed that the scaled up intervention will have the same effectiveness as reported in the meta-analysis of high coverage trials i.e. a 33% reduction in neonatal mortality (RR=0.67). Secondly, to estimate a lower bound, we assumed a 30% loss of effectiveness when the intervention is implemented at scale.

Table A3 summarises the population data used for these calculations.

Table A3: Scale up assumption data

|  | **India** | **Nepal** | **Bangladesh** | **Malawi** |
| --- | --- | --- | --- | --- |
| Rural population | 885,393,934 | 3,477,494 | 105,861,481 | 15,114,787 |
| Annual number of rural births* | 18,327,654 | 617,458 | 2,413,642 | 492,742 |
| Neonatal mortality rate in rural areas (per 1000 live births) | 33.1 | 24 | 31 | 27 |
| Rate of skilled attendance in rural regions (%) | 78.0 | 46.8 | 35.6 | 88.9 |

Sources: 2016 figures or earlier from UNICEF Global databases and World Development Indicators. *Authors’ estimate.

**References**

UNICEF. Unicef Global Databases [Internet]. Available from: data.unicef.org/maternal-health/delivery-care. Cited September 2014.

THE WORLD BANK. World development indicators [Internet]. Available from: data.worldbank.org/indicator/SP.RUR.TOTL/countries. Cited January 2019.
