# Supplementary material for: Participatory learning and action cycles with women’s groups to prevent neonatal death in low-resource settings: A multi-country comparison of cost-effectiveness and affordability
Source: Health Policy Plan. 2020 Oct 21;35(10):1280–9. doi: 10.1093/heapol/czaa081 (PMC7886438; doi:10.1093/heapol/czaa081)
Supplement: czaa081_Supplementary_Data [file czaa081_supplementary_data.zip › Appendix 4 Annual aggregate costs.docx]

Appendix 4: Annual aggregate costs

This Appendix presents annual aggregate costs for each trial. All figures are in 2016 INT$. A 3% annual discount rate has been applied with the base year as the first year for each trial. Staff, Materials, Other recurrent costs and Capital costs sum up to total costs. Summing up Start-up and Implementation gives the same totals.

Table A4-1: India (Ekjut)

| **Cost category** | **2004** | **2005** | **2006** | **2007** | **2008** |  |
| --- | --- | --- | --- | --- | --- | --- |
| Staff | 45172 | 123506 | 155566 | 135447 | 98018 |  |
| Materials | 1295 | 8656 | 9555 | 8284 | 2425 |  |
| Other recurrents | 3354 | 19918 | 24950 | 34936 | 16883 |  |
| Capital | 4460 | 24492 | 40404 | 26476 | 13415 |  |
| Start-up | 54281 | 141258 | 46095 | 41029 | 26148 |  |
| Implementation | 0 | 35314 | 184380 | 164114 | 104593 |  |

Table A4-2: Nepal

| **Cost category** | **1999** | **2000** | **2001** | **2002** | **2003** |  |
| --- | --- | --- | --- | --- | --- | --- |
| Staff | 56005 | 326244 | 310795 | 298864 | 207282 |  |
| Materials | 1266 | 7198 | 9987 | 7301 | 3483 |  |
| Other recurrents | 8191 | 46560 | 31814 | 31883 | 20642 |  |
| Capital | 5985 | 34018 | 37460 | 65303 | 45741 |  |
| Start-up | 71446 | 414019 | 32505 | 0 | 0 |  |
| Implementation | 0 | 0 | 357551 | 403351 | 277148 |  |

Table A4-3: Bangladesh I

| **Cost category** | **2002** | **2003** | **2004** | **2005** | **2006** | **2007** |
| --- | --- | --- | --- | --- | --- | --- |
| Staff | 93846 | 190868 | 207673 | 94795 | 118611 | 134741 |
| Materials | 0 | 0 | 0 | 0 | 0 | 0 |
| Other recurrents | 46045 | 95184 | 93064 | 32255 | 41001 | 42626 |
| Capital | 27884 | 50091 | 54506 | 21817 | 19137 | 23807 |
| Start-up | 167774 | 336142 | 177621 | 29773 | 0 | 0 |
| Implementation | 0 | 0 | 177621 | 119094 | 178749 | 201174 |

Table A4-4: Bangladesh II (Scale-up phase only*)

| **Cost category** | **2008** | **2009** | **2010** | **2011** |  |  |
| --- | --- | --- | --- | --- | --- | --- |
| Staff | 249112 | 428787 | 478082 | 239795 |  |  |
| Materials | 0 | 2090 | 20206 | 6941 |  |  |
| Other recurrents | 65648 | 65178 | 90536 | 57272 |  |  |
| Capital | 66354 | 89541 | 80444 | 23889 |  |  |
| Start-up | 190557 | 119048 | 49731 | 0 |  |  |
| Implementation | 190557 | 466548 | 619538 | 327897 |  |  |

Notes to Table: *See paper for a description of how these costs were used for Bangladesh II-Modelled.

Table A4-5: Malawi-MaiMwana

| **Cost category** | **2004** | **2005** | **2006** | **2007** | **2008** | **2009** |
| --- | --- | --- | --- | --- | --- | --- |
| Staff | 71012 | 113369 | 124258 | 122619 | 118863 | 9257 |
| Materials | 4810 | 8120 | 1387 | 6800 | 13453 | 2245 |
| Other recurrents | 19674 | 31139 | 54240 | 36735 | 46094 | 2872 |
| Capital | 15508 | 24782 | 21820 | 24796 | 26378 | 4273 |
| Start-up | 111004 | 18099 | 39292 | 27742 | 33969 | 1863 |
| Implementation | 0 | 159311 | 162413 | 163207 | 170818 | 16784 |

Table A4-6: Malawi-MaiKhanda

| **Cost category** | **2008** | **2009** | **2010** | **2011** |  |  |
| --- | --- | --- | --- | --- | --- | --- |
| Staff | 402315 | 376597 | 517592 | 269088 |  |  |
| Materials | 41508 | 16244 | 16386 | 29283 |  |  |
| Other recurrents | 158890 | 237840 | 231546 | 152430 |  |  |
| Capital | 191686 | 149518 | 113159 | 160761 |  |  |
| Start-up | 391292 | 9614 | 32109 | 11672 |  |  |
| Implementation | 403107 | 770585 | 846573 | 599890 |  |  |
