# Supplementary material for: Participatory learning and action cycles with women’s groups to prevent neonatal death in low-resource settings: A multi-country comparison of cost-effectiveness and affordability
Source: Health Policy Plan. 2020 Oct 21;35(10):1280–9. doi: 10.1093/heapol/czaa081 (PMC7886438; doi:10.1093/heapol/czaa081)
Supplement: czaa081_Supplementary_Data [file czaa081_supplementary_data.zip › Appendix 5 Comparison with previously published results.docx]

Appendix 5: Comparison with previously published results

In Table A5 we report previously published cost-effectiveness ratios from the trial-specific papers and the meta-analysis paper, alongside the base case results reported in the present paper. Table A5 thus complements Table 1: *Summary of previously published cost-effectiveness evidence*. Some trial-specific papers reported more than one result; here, we report the result that is most comparable with the cost-effectiveness ratios reported in the present paper.

Table A5: Summary of previously published cost-effectiveness ratios

| **Trial** | **Cost-effectiveness ratio reported in trial-specific paper** | **Cost-effectiveness ratio reported in systematic review** | **Cost-effectiveness ratio reported in this paper** |
| --- | --- | --- | --- |
| **Currency and reporting year** | **Specified below** | **2011 INT$** | **2016 INT$** |
| India (Ekjut) | $33 per neonatal LYS (2007 USD) | $91 per neonatal YLL averted | $135 per neonatal LYS |
| India (Mumbai) | - | - | - |
| Nepal | $211 per neonatal LYS (2003 USD) | $753 per neonatal YLL averted | $1,627 per neonatal LYS |
| Bangladesh I | - | - | $787 per neonatal LYS |
| Bangladesh II | $393 per neonatal LYS (2011 USD) | $650 per neonatal YLL averted | $634 per neonatal LYS |
| Malawi-MaiMwana | $114 per LYS (neonatal + maternal) (2011 USD) | $577 per neonatal YLL averted | $768 per neonatal LYS |
| Malawi-MaiKhanda | $79 per DALY averted (2013 INT$) | - | $285 per neonatal LYS |

Notes to Table A: LYS – life-year saved. YLL – year of life lost. DALY – disability-adjusted life-year.
