# Supplementary material for: Participatory learning and action cycles with women’s groups to prevent neonatal death in low-resource settings: A multi-country comparison of cost-effectiveness and affordability
Source: Health Policy Plan. 2020 Oct 21;35(10):1280–9. doi: 10.1093/heapol/czaa081 (PMC7886438; doi:10.1093/heapol/czaa081)
Supplement: czaa081_Supplementary_Data [file czaa081_supplementary_data.zip › Appendix 6 Associations between unit costs and cost-effectiveness ratios with coverage, scale, and intensity of the intervention.docx]

# Appendix 6: Associations between unit costs and cost-effectiveness ratios with coverage, scale, and intensity of the intervention

This Appendix presents scatter plots and correlation coefficients for our analyses of possible reasons for variation in cost-effectiveness ratios across countries.

We first examined the correlation between the cost-effectiveness ratio and effectiveness on the one hand, and unit costs on the other hand. As expected, the cost-effectiveness ratio tends to be smaller the larger the number of life-years saved (Figure A6-1). However, this relationship between effectiveness and the cost-effectiveness ratio was not statistically significant in this sample (r=-0.71, P=0.11). In contrast, a statistically significant, strong, positive association between cost per life-birth and the cost-effectiveness ratio was found (r=0.90, P=0.01; see Figure 2 in the main manuscript).

**Figure A6-1: Association between the cost-effectiveness ratio and effectiveness**

We then assessed whether intervention coverage, which was previously found to be a significant determinant of the effectiveness of women’s groups ([Prost et al., 2013](#_ENREF_1)), was associated with unit costs and cost-effectiveness ratios. We found no evidence in our data that the proportion of pregnant women attending groups would be associated with either cost per live-birth (r=0.45, P=0.37; Figure A6-2) or the cost-effectiveness ratio (r=0.20, P=0.71; Figure A6-3).

**Figure A6-2: Association between unit costs and intervention coverage**


**Figure A6-3: Association between the cost-effectiveness ratio and intervention coverage**

The fact that differences in intervention coverage do not explain differences in cost and, especially, cost-effectiveness ratios is apparent when comparing countries with similar coverage. The two countries with low coverage (Bangladesh-I and Malawi-MaiKhanda; 3% and 10% respectively) had similar unit costs but different cost-effectiveness ratios. Across three countries with similar, higher levels of coverage (India, Nepal, and Bangladesh-II; 36-37%), both unit costs and cost-effectiveness ratios were widely different.

Third, we examined whether the scale of delivery could explain some of the variation in cost and cost-effectiveness across countries. Figure A6-4 suggests positive economies of scale for potential beneficiaries: cost per live-birth decreases as the number of live-births increases. However, this association was not statistically significant (r=-0.54, P=0.26). We similarly found no statistically significant indications of economies of scale for total population size (r=-0.46; P=0.35).

**Figure A6-4: Economies of scale for new-borns**

Positive economies of scale suggests that the cost-effectiveness ratio may be smaller, the larger the scale of delivery. However, we found no statistically significant association between cost per neonatal life-year saved, and either the number of live-births (r=-0.50 P=0.31; Figure A6-5) or total population size (r=-0.44; P=0.38).

**Figure A6-5: Association between the cost-effectiveness ratio and the number of live-births**

Finally, there was no evidence that intervention intensity, indicated by the number of women’s groups, was associated with either cost per live birth (r=-0.09; P=0.86) or the cost-effectiveness ratio (r=-0.32; P=0.53).

**References**

PROST, A., COLBOURN, T., SEWARD, N., AZAD, K., COOMARASAMY, A., COPAS, A., HOUWELING, T. A. J., FOTTRELL, E., KUDDUS, A., LEWYCKA, S., MACARTHUR, C., MANANDHAR, D., MORRISON, J., MWANSAMBO, C., NAIR, N., NAMBIAR, B., OSRIN, D., PAGEL, C., PHIRI, T., PULKKI-BRÄNNSTRÖM, A.-M., ROSATO, M., SKORDIS-WORRALL, J., SAVILLE, N., MORE, N. S., SHRESTHA, B., TRIPATHY, P., WILSON, A. & COSTELLO, A. 2013. Women's groups practising participatory learning and action to improve maternal and newborn health in low-resource settings: a systematic review and meta-analysis. *The Lancet,* 381**,** 1736-1746.
