# Supplementary material for: Participatory learning and action cycles with women’s groups to prevent neonatal death in low-resource settings: A multi-country comparison of cost-effectiveness and affordability
Source: Health Policy Plan. 2020 Oct 21;35(10):1280–9. doi: 10.1093/heapol/czaa081 (PMC7886438; doi:10.1093/heapol/czaa081)
Supplement: czaa081_Supplementary_Data [file czaa081_supplementary_data.zip › Table 1.docx]

Table 1: Summary of previously published cost-effectiveness evidence

| **Trial** | **Cost-effectiveness analysed in trial-specific paper** | **Cost-effectiveness included in systematic review (**[**Prost et al., 2013**](#_ENREF_25)**)** | **Cost data re-analysed in this paper** |
| --- | --- | --- | --- |
| India (Ekjut) | Yes ([Tripathy et al., 2010](#_ENREF_28)) | Yes | Yes |
| India (Mumbai) | No | No | No |
| Nepal | Yes ([Borghi et al., 2005](#_ENREF_5)) | Yes | Yes |
| Bangladesh I | No | No | Yes |
| Bangladesh II | Yes ([Fottrell et al., 2013](#_ENREF_15)) | Yes | Yes |
| Malawi-MaiMwana | Yes ([Lewycka et al., 2013](#_ENREF_19)) | Yes | Yes |
| Malawi-MaiKhanda | Yes ([Tim Colbourn et al., 2015](#_ENREF_9)) | No | Yes |
