# Supplementary material for: Participatory learning and action cycles with women’s groups to prevent neonatal death in low-resource settings: A multi-country comparison of cost-effectiveness and affordability
Source: Health Policy Plan. 2020 Oct 21;35(10):1280–9. doi: 10.1093/heapol/czaa081 (PMC7886438; doi:10.1093/heapol/czaa081)
Supplement: czaa081_Supplementary_Data [file czaa081_supplementary_data.zip › Table 2.docx]

Table 2: Description and comparison of the interventions

| **Characteristic** | **India** | **Nepal** | **Bangladesh**  **I** | **Bangladesh II** | **Malawi - MaiMwana** | **Malawi - MaiKhanda** |
| --- | --- | --- | --- | --- | --- | --- |
| Intervention period (months) | 36 | 24 | 35 | 30 | 36 | 27 |
| Cost-effectiveness time horizon (months) | 49 | 48 | 66 | 42 | 55 | 48 |
| Intervention area population | 114,141 | 86,704 | 229,195 | 243,341 | 94,992¶ | 1,200,000†¶ |
| Live births‡ | 9,469 | 2,899 | 15,153 | 8,819 | 9,174¶ | 100,000†¶ |
| Women’s groups | 244 | 111 | 162 | 810 | 207¶ | 729¶ |
| Meetings per group (average) | 20 | 10 | 20 | 24 | 20 | 16 |
| Intervention coverage (%)§ | 37 | 37 | 3 | 36 | 51 | 10 |

Notes to Table 2: The figures are based on published papers ([Azad et al., 2010](#_ENREF_1); [Borghi et al., 2005](#_ENREF_5); [T Colbourn, Nambiar, & Costello, 2013](#_ENREF_7); [Tim Colbourn et al., 2013](#_ENREF_8); [Tim Colbourn et al., 2015](#_ENREF_9); [Fottrell et al., 2013](#_ENREF_15); [Lewycka et al., 2013](#_ENREF_19); [Manandhar et al., 2004](#_ENREF_20); [More et al., 2012](#_ENREF_21); [Prost et al., 2013](#_ENREF_25); [Tripathy et al., 2010](#_ENREF_28)). †Estimated. Birth and death surveillance data were captured on an estimated 9% of total births. ‡During the intervention period. §Percentage of pregnant women who reported having attended at least one women’s group meeting ([2013](#_ENREF_25)). ¶Full intervention area. Subsequently we use figures relating to half this area (women’s groups only arm), as described in the Methods section.
