# Supplementary material for: Participatory learning and action cycles with women’s groups to prevent neonatal death in low-resource settings: A multi-country comparison of cost-effectiveness and affordability
Source: Health Policy Plan. 2020 Oct 21;35(10):1280–9. doi: 10.1093/heapol/czaa081 (PMC7886438; doi:10.1093/heapol/czaa081)
Supplement: czaa081_Supplementary_Data [file czaa081_supplementary_data.zip › Table 3.docx]

Table 3: Cost description of the women’s groups intervention (2016 INT$)

|  | **India** | **Nepal** | **Bangla-desh I** | **Bangladesh II-Modelled** | **Malawi- MaiMwana**† | **Malawi- MaiKhanda** † | ***Mean*** |
| --- | --- | --- | --- | --- | --- | --- | --- |
| Total cost | 797,212 | 1,556,020 | 1,387,949 | 2,237,115 | 904,504 | 3,064,842 | *1,657,941* |
| Start-up cost | 308,810 | 517,970 | 711,312 | 1,094,497 | 231,969 | 444,687 | *551,541* |
| Annual total cost‡ | 195,236 | 389,005 | 252,354 | 639,176 | 197,346 | 766,211 | *406,555* |
| Annual cost of implementa­tio­n§ | 119,609 | 259,512 | 123,025 | 326,462 | 146,735 | 655,039 | *271,730* |
|  |  |  |  |  |  |  |  |

Notes: †Women’s groups only arm (see Methods). ‡Averaged over the cost-effectiveness time horizon (see Table 2). §Averaged over the intervention period (see Table 2).
