# Supplementary material for: Participatory learning and action cycles with women’s groups to prevent neonatal death in low-resource settings: A multi-country comparison of cost-effectiveness and affordability
Source: Health Policy Plan. 2020 Oct 21;35(10):1280–9. doi: 10.1093/heapol/czaa081 (PMC7886438; doi:10.1093/heapol/czaa081)
Supplement: czaa081_Supplementary_Data [file czaa081_supplementary_data.zip › Table 4.docx]

Table 4: Unit costs of the intervention (2016 INT$)

| **Unit costs** | **India** | **Nepal** | **Bangladesh I** | **Bangladesh II-Modelled** | **Malawi-MaiMwana** | **Malawi-MaiKhanda** | ***Mean*** |
| --- | --- | --- | --- | --- | --- | --- | --- |
| Cost per live-birth | 84 | 537 | 92 | 254 | 193 | 61 | *203* |
| Annual cost per group | 800 | 3,505 | 1,558 | 789 | 1,907 | 2,102 | *1,777* |
| Annual cost per person (all ages) | 1.7 | 4.5 | 1.1 | 2.6 | 4.2 | 1.3 | *2.6* |
