# Supplementary material for: Participatory learning and action cycles with women’s groups to prevent neonatal death in low-resource settings: A multi-country comparison of cost-effectiveness and affordability
Source: Health Policy Plan. 2020 Oct 21;35(10):1280–9. doi: 10.1093/heapol/czaa081 (PMC7886438; doi:10.1093/heapol/czaa081)
Supplement: czaa081_Supplementary_Data [file czaa081_supplementary_data.zip › Table 5.docx]

Table 5: Effectiveness and cost-effectiveness of women’s groups (2016 INT$)

|  | **India** | **Nepal** | **Bangladesh I** | **Bangladesh II-Modelled** | **Malawi-MaiMwana** | **Malawi- MaiKhanda** | ***Mean*** |
| --- | --- | --- | --- | --- | --- | --- | --- |
| Neonatal deaths averted | 191 | 31 | 57 | 115 | 38 | 350 | *130* |
| Neonatal life-years saved† | 5,887 | 956 | 1,763 | 3,531 | 1,178 | 10,770 | *4,014* |
| Cost per neonatal life-year saved | $135 | $1,627 | $787 | $634 | $768 | $285 | *$706* |
| GDP per capita, PPP for 2016‡ | $6,572 | $2,468 | $3,581 | $3,581 | $1,169 | $1,169 | *N/A* |

Notes to Table: †Discounted at 3%. ‡Threshold value for “very cost-effective” interventions.
