# Supplementary material for: Participatory learning and action cycles with women’s groups to prevent neonatal death in low-resource settings: A multi-country comparison of cost-effectiveness and affordability
Source: Health Policy Plan. 2020 Oct 21;35(10):1280–9. doi: 10.1093/heapol/czaa081 (PMC7886438; doi:10.1093/heapol/czaa081)
Supplement: czaa081_Supplementary_Data [file czaa081_supplementary_data.zip › Table 6.docx]

Table 6: Cost and affordability of scaling up to national delivery

| **Description** | **India** | **Nepal** | **Bangladesh†** | **Malawi‡** |
| --- | --- | --- | --- | --- |
| Average annual cost (million $) | 1,514 | 105 | 278 | 41 |
| % Total health expenditure | 0.37 | 2.54 | 1.70 | 1.70 |
| % Government health expenditure | 1.23 | 6.30 | 6.10 | 3.23 |
| % GDP | 0.02 | 0.15 | 0.05 | 0.19 |

Notes to Table: **†**Data and assumptions for Bangladesh II-Modelled were used here. ‡Mean value of unit costs for Malawi-MaiMwana and Malawi-MaiKhanda were used here.
