# Supplementary material for: Participatory learning and action cycles with women’s groups to prevent neonatal death in low-resource settings: A multi-country comparison of cost-effectiveness and affordability
Source: Health Policy Plan. 2020 Oct 21;35(10):1280–9. doi: 10.1093/heapol/czaa081 (PMC7886438; doi:10.1093/heapol/czaa081)
Supplement: czaa081_Supplementary_Data [file czaa081_supplementary_data.zip › Table 7.docx]

Table 7: Estimated number of neonatal lives saved by scaling up women’s groups

| **Countries** | **Assuming NO loss of effectiveness at scale** | | **Assuming 30% loss of effectiveness at scale** | |
| --- | --- | --- | --- | --- |
|  | No. neonatal lives saved | % of total neonatal deaths | No. neonatal lives saved | % of total neonatal deaths |
| India | 42,780 | 5% | 29,946 | 3% |
| Nepal | 2,527 | 15% | 1,769 | 11% |
| Bangladesh | 15,445 | 14% | 10,812 | 10% |
| Malawi | 473 | 3% | 331 | 2% |
| **Total** | 61,226 | 9% | 42,858 | 7% |
