# Supplementary material for: Participatory learning and action cycles with women’s groups to prevent neonatal death in low-resource settings: A multi-country comparison of cost-effectiveness and affordability
Source: Health Policy Plan. 2020 Oct 21;35(10):1280–9. doi: 10.1093/heapol/czaa081 (PMC7886438; doi:10.1093/heapol/czaa081)
Supplement: czaa081_Supplementary_Data [file czaa081_supplementary_data.zip › Table 8.docx]

Table 8: Results of one-way sensitivity analyses on cost per life-year saved (2016 INT$)

| **Scenarios/ Parameters** | **India** | **Nepal** | **Bangladesh I** | **Bangladesh II-Modelled** | **Malawi-MaiMwana** | **Malawi-MaiKhanda** |
| --- | --- | --- | --- | --- | --- | --- |
| **Base case scenario** | 135 | 1,627 | 787 | 634 | 768 | 285 |
| GDP per capita | 6,572 | 2,468 | 3,581 | 3,581 | 1,169 | 1,169 |
| **Health outcomes (Base case neonatal life-years saved only)** | | | | | | |
| Add maternal life-years saved**†** | 123 | 1,325 | N/A | 610 | 576 | 268 |
| **Start-up costs (Base case 100%)** | | | | | | |
| Reduce start-up costs by 50% | 109 | 1,356 | 586 | 479 | 670 | 264 |
| **Joint cost allocation rules‡** | | | | | | |
| Base case allocation rule (%) | 29-36% | N/A | 40% | 40% | 25% | 30-35% |
| -10% points | 126 | N/A | 713 | 614 | 689 | 242 |
| +10% points | 145 | N/A | 862 | 653 | 847 | 328 |
| **Inclusion of implementation costs in factorial trials (Base case 50%)** | | | | | | |
| 33% costs included | N/A | N/A | N/A | N/A | 574 | 202 |
| 75% costs included | N/A | N/A | N/A | N/A | 1,054 | 406 |
| **Discount rate (Base case 3% both costs and life-years)** | | | | | | |
| Costs 0%, life-years 0% | 52 | 623 | 302 | 234 | 295 | 106 |
| Costs 6%, life-years 3% | 127 | 1,526 | 737 | 616 | 720 | 276 |

Notes to Table: **†**Discounted at 3%. ‡It was not possible to run this analysis for Nepal.
