# Supplementary figures and images for: Participatory learning and action cycles with women’s groups to prevent neonatal death in low-resource settings: A multi-country comparison of cost-effectiveness and affordability
Source: Health Policy Plan. 2020 Oct 21;35(10):1280–9. doi: 10.1093/heapol/czaa081 (PMC7886438; doi:10.1093/heapol/czaa081)

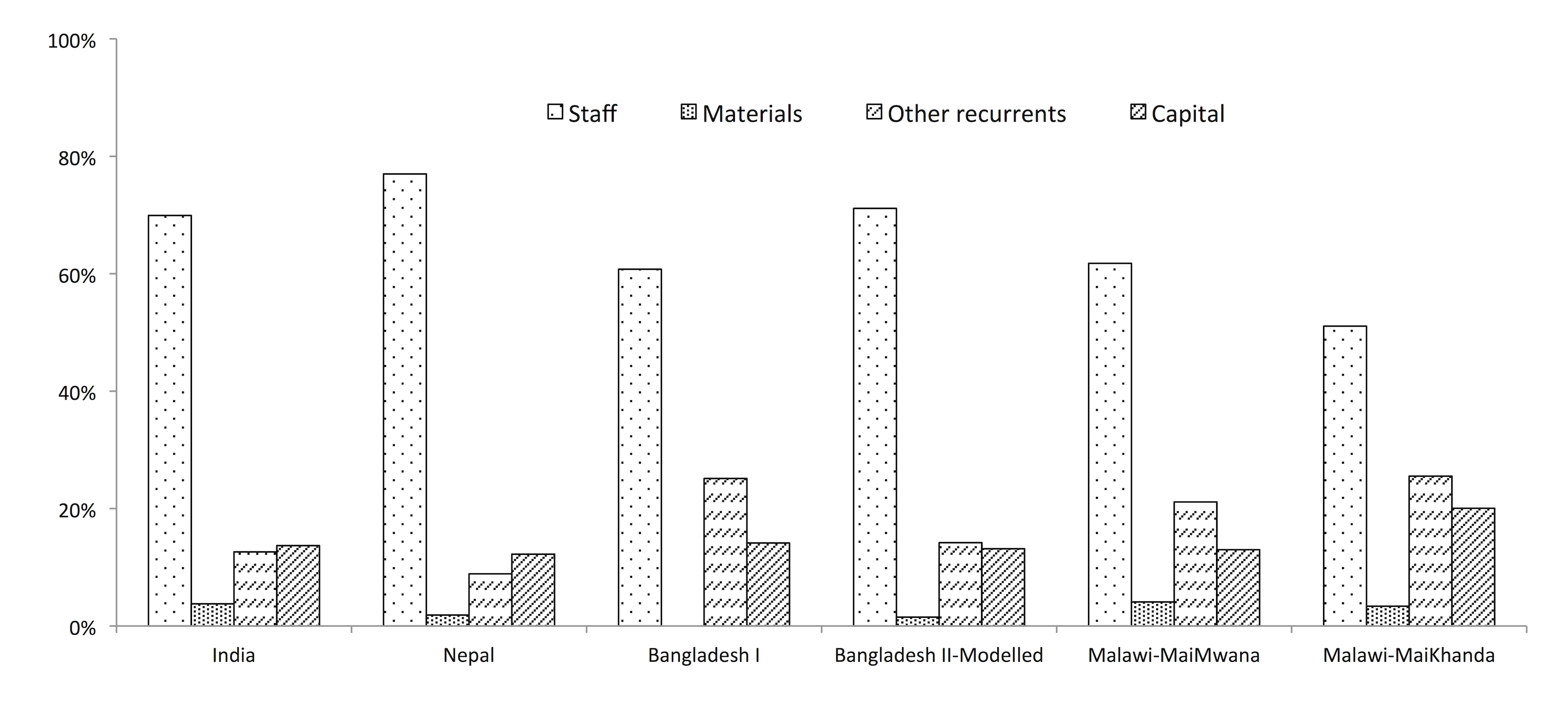

Supplement: czaa081_Supplementary_Data [file czaa081_supplementary_data.zip › Figure 1 Components of total cost SMALL.tiff]

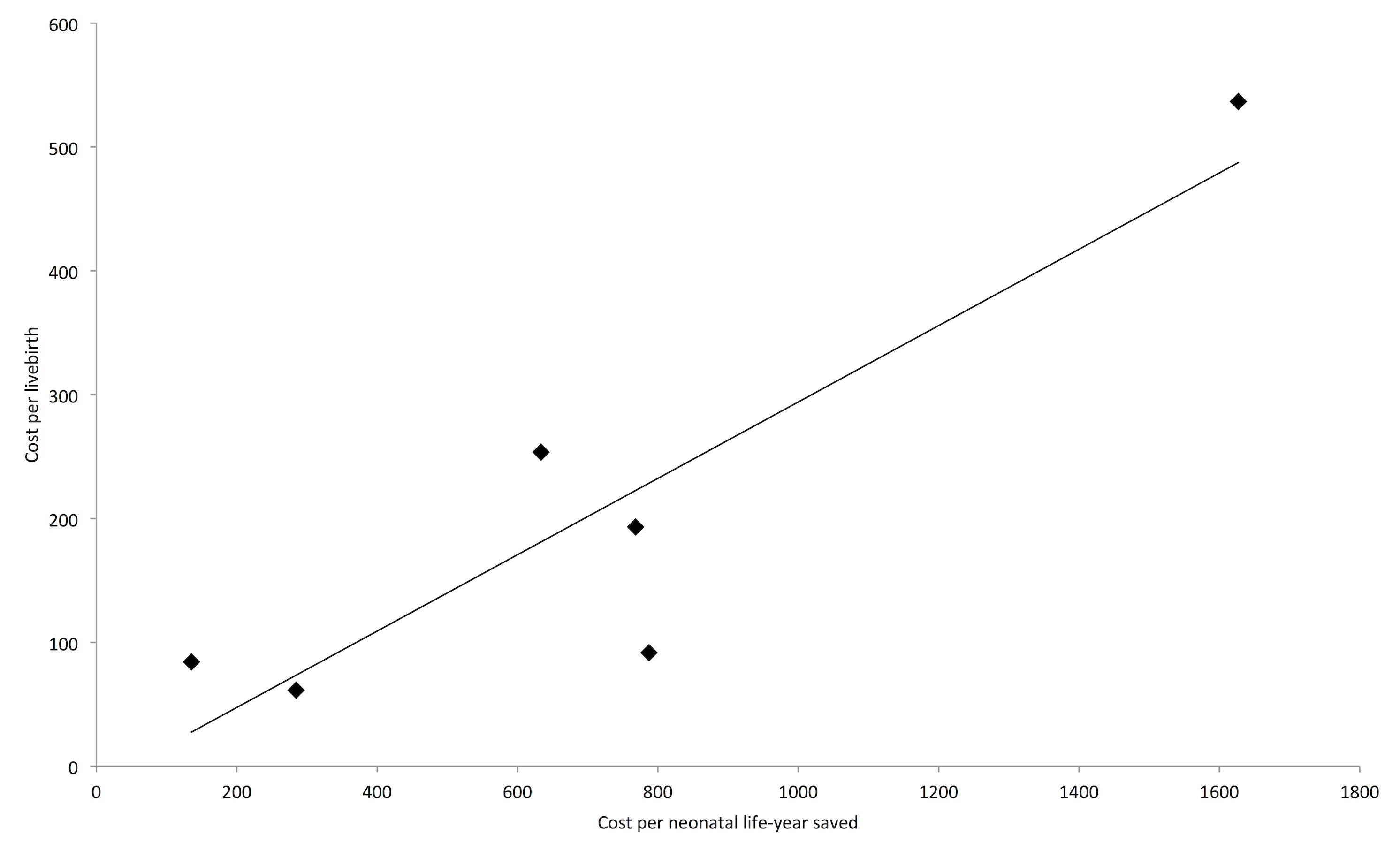

Supplement: czaa081_Supplementary_Data [file czaa081_supplementary_data.zip › Figure 2 Association between the cost-effectiveness ratio and unit costs SMALL.tiff]
